# UV-Vis-Induced Degradation of Phenol over Magnetic Photocatalysts Modified with Pt, Pd, Cu and Au Nanoparticles

**DOI:** 10.3390/nano8010028

**Published:** 2018-01-07

**Authors:** Izabela Wysocka, Ewa Kowalska, Konrad Trzciński, Marcin Łapiński, Grzegorz Nowaczyk, Anna Zielińska-Jurek

**Affiliations:** 1 Faculty of Chemistry, Gdansk University of Technology, 80-233 Gdansk, Poland; iziawww@gmail.com (I.W.); kontrzci@pg.edu.pl (K.T.); 2 Institute for Catalysis (ICAT), Hokkaido University, Sapporo 001-0021, Japan; kowalska@cat.hokudai.ac.jp; 3 Faculty of Applied Physics and Mathematics, Gdansk University of Technology, 80-233 Gdansk, Poland; marcin.lapinski@pg.edu.pl; 4 NanoBioMedical Center, Adam Mickiewicz University, 61-614 Poznan, Poland; nowag@amu.edu.pl

**Keywords:** heterogeneous photocatalysis, magnetic photocatalysts, modification of titanium(IV) oxide, mechanism of degradation, noble metal nanoparticles, phenol

## Abstract

The combination of TiO_2_ photocatalyst and magnetic oxide nanoparticles enhances the separation and recoverable properties of nanosized TiO_2_ photocatalyst. Metal-modified (Me = Pd, Au, Pt, Cu) TiO_2_/SiO_2_@Fe_3_O_4_ nanocomposites were prepared by an ultrasonic-assisted sol-gel method. All prepared samples were characterized by X-ray powder diffraction (XRD) analysis, Brunauer-Emmett-Teller (BET) method, X-ray photoelectron spectroscopy (XPS), scanning transmission electron microscopy (STEM), Mott-Schottky analysis and photoluminescence spectroscopy (PL). Phenol oxidation pathways of magnetic photocatalysts modified with Pt, Pd, Cu and Au nanoparticles proceeded by generation of reactive oxygen species, which oxidized phenol to benzoquinone, hydroquinone and catechol. Benzoquinone and maleic acid were products, which were determined in the hydroquinone oxidation pathway. The highest mineralization rate was observed for Pd-TiO_2_/SiO_2_@Fe_3_O_4_ and Cu-TiO_2_/SiO_2_@Fe_3_O_4_ photocatalysts, which produced the highest concentration of catechol during photocatalytic reaction. For Pt-TiO_2_/SiO_2_@Fe_3_O_4_ nanocomposite, a lack of catechol after 60 min of irradiation resulted in low mineralization rate (CO_2_ formation). It is proposed that the enhanced photocatalytic activity of palladium and copper-modified photocatalysts is related to an increase in the amount of adsorption sites and efficient charge carrier separation, whereas the keto-enol tautomeric equilibrium retards the rate of phenol photomineralization on Au-TiO_2_/SiO_2_@Fe_3_O_4_. The magnetization hysteresis loop indicated that the obtained hybrid photocatalyst showed magnetic properties and therefore could be easily separated after treatment process.

## 1. Introduction

Degussa (Evonik) TiO_2_ P25 consisting of a mixture of anatase (∼78%), rutile (∼14%) phases and a minor amount of amorphous phase (∼8%) is a well-known commercial material frequently used to oxidize organic and inorganic compounds in air and water due to its strong oxidative ability and long-term photo-stability [1,2]. The photocatalytic activity of TiO_2_ P25 is affected by light absorption, charge creation/recombination rate and surface activity. However, based on practicality, TiO_2_-based photocatalysis has some technical limitations that impede its commercialization. The first one is the separation and recovery of fine TiO_2_ particles after purification process. The sedimentation of photocatalyst is usually insufficient. Even when the photocatalyst flocculates, there is often some residue of TiO_2_ nanoparticles in the supernatant. In this regard, the combination of photocatalyst and magnetic particles is a suitable solution for the separation issue, allowing for a complete recovery of TiO_2_-based nanomaterial [3]. In recent years, the incorporation of magnetic particles into TiO_2_-based photocatalysts has attracted considerable attention. However, some shortcomings of magnetic photocatalysts have been also reported. For example, Beydoun et al. stated that direct contact between TiO_2_ and iron oxides (e.g., Fe_3_O_4_, Fe_2_O_3_) could decrease photocatalytic activity and reduce the magnetic properties due to photo-dissolution of iron phase induced by the TiO_2_ layer [4,5]. In this regard, the introduction of a silica intermediate passive layer could prevent an injection of charge carriers from titania shell into the iron oxide core and the subsequent leaching of iron ions into the solution [5]. The functionalization of the magnetic core consisting of magnetite [6], maghemite [7], hematite [8], CoFe_2_O_4_ [9], ZnFe_2_O_4_ spinel ferrites [10], BaFe_12_O_19_ hexagonal ferrites [9], bimetallic nanoparticles of Fe-Ni, Co-Fe, Mn-Zn, Ni-Cu-Zn [11,12] with TiO_2_ and intermediate SiO_2_ passive layer has recently been investigated.

However, there are very few reports in the literature regarding activation of magnetic photocatalysts under visible light. TiO_2_-based photocatalysts require an excitation wavelength that falls in the ultraviolet (UV) region. Since UV represents a small part (ca. 3–5%) of the solar radiation, there is a need to design new photocatalysts with the ability to work under UV-visible light irradiation to provide better utilization of solar energy and to generate a greater number of photo-excited charge carriers. Magnetically-recoverable nitrogen-doped TiO_2_-based photocatalysts were studied for the degradation of phenol [13,14], p-nitrophenol [15], methyl orange [16], ibuprofen, benzochinone and carbamazepine [17]. Several studies have been reported using noble metals as the surface modifiers deposited on TiO_2_-based magnetic photocatalysts. Noble metals such as silver, gold, platinum, and palladium with a Fermi level lower than the electron affinity of TiO_2_ enhance electron-hole separation and possess the ability to absorb visible light due to localized surface plasmon resonance (LSPR) properties [18]. Magnetic photocatalysts modified with Ag, Pt, Ag-Pd or Au particles have been studied in the photocatalytic removal of chlorophenol [19], and mainly in the reaction of organic dye removal such as rhodamine B [20,21] and methylene blue [12,22]. Although the effect of TiO_2_ modified with noble and semi-noble metal nanoparticles in photo-oxidation of chlorophenol [19] and dyes [20] for magnetic photocatalysts and chlorophenol [23,24], toluene [25,26,27], pesticides [28,29], carbonic acid [30,31], and dyes [32,33] for metal-modified TiO_2_ photocatalysts has been proved, the influence of the photocatalyst’s structure on the photocatalytic activity and the mechanism of photocatalytic reaction still needs clarification. Reaction intermediates and product formation pathways depend on the physicochemical properties of the photocatalyst as well as experimental conditions, e.g., reaction environment, aeration, concentration and type of organic compounds, irradiation source and temperature [34,35,36,37]. Therefore, the direct comparison of experimental results with the literature on the effect of noble metal deposition on the photocatalytic activity is difficult.

In this regard, the aim of the present study is the determination of the phenol photo-oxidation mechanism in the presence of magnetically-separable noble metal-modified (Me = Pd, Au, Pt, Cu) Me-TiO_2_/SiO_2_@Fe_3_O_4_ photocatalysts. The combination of TiO_2_ and magnetic oxide nanoparticles enhances the separation and recoverable properties of nanosized TiO_2_ photocatalyst under a magnetic field. Moreover, the metals deposited on TiO_2_ serve as a source of electron traps, which can increase the lifetime of separated electron-hole pairs, and thus enhance the efficiency and product selectivity for organic compound photodegradation [38]. However, the method of photocatalyst modification, i.e., surface or bulk, may also lead to the changes in reaction mechanism.

Phenol is an intermediate product aromatic hydrocarbon oxidation pathway; thus, it is commonly used as a model organic compound for advanced wastewater treatment reactions. TiO_2_ photocatalytic degradation of phenolic compounds has been intensively studied, and focus was put mainly on preparation and modification of new photocatalytic materials. The main phenol degradation intermediates are hydroquinone, benzoquinone, catechol, resorcinol, salicylic acid, 1,2,3-benzotriol, glycerol, muconic acid, maleic acid, oxalic acid and formaldehyde [39], which are generated under aerobic conditions. Su et al. [37] proposed a mechanism of phenol degradation over Au/Pd bimetallic particle-modified TiO_2_ as a result of nonselective hydroxyl radical attack, leading to the generation of p-benzoquinone and hydroquinone. For commercial titanium(IV) oxide, P25, it was observed that only hydroquinone appeared in substantial amounts. Catechol presence was also detected; however, the concentration was negligible. It was found that, during phenol photodegradation, the hydroquinone pathway was a limiting step. Zhang et al. [40] found that catechol and hydroquinone were the main products generated immediately during phenol photocatalytic oxidation. The next step was further hydroxylation of the aromatic ring leading to the occurrence of a trisubstituted compound such as pyrogallol. They stated that the final step of phenol oxidation should have been organic acids; however, their presence was not confirmed. In some studies [41], it has been proposed that oligomerization or polimerization reactions occurred, yielding non-oxidizable compounds.

In the present study, to provide insight into the mechanism of phenol degradation, the determination of oxidative species participating in the degradation mechanism has been investigated by reference experiments in the presence of scavengers (h_vb_^+^, e_cb_^−^ and free radicals). The correlation between surface properties of magnetic nanocomposites modified with Au, Pd, Pt or Cu and photocatalytic activities has been studied. The effect of the noble metal loadings on phenol oxidation pathways via various intermediates, and finally its mineralization, measured as total organic carbon (TOC) reduction, has been investigated for the first time.

## 2. Results

### 2.1. Characterization of Nanocomposites

**Figure 1:**
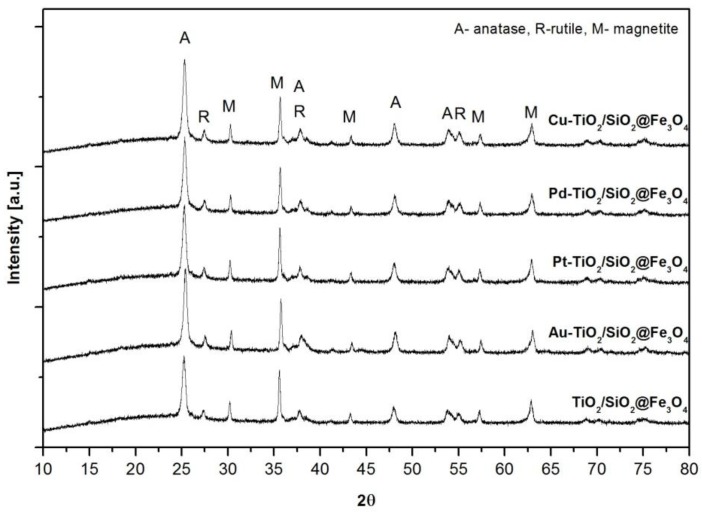
X-ray diffraction (XRD) patterns of Me-TiO_2_/SiO_2_@Fe_3_O_4_ nanocomposites.

X-ray diffraction (XRD) patterns of TiO_2_/SiO_2_@Fe_3_O_4_ samples modified with nanoparticles of Cu, Pt, Pd and Au are shown in Figure 1. All prepared samples revealed high crystallinity of cubic spinel Fe_3_O_4_, anatase and rutile phases.

The estimated values of phase content and crystallite sizes are presented in Table 1. The average primary size of anatase crystals was about 20 ± 0.5 nm, whereas, for the rutile phase, it was 30 ± 1 nm. Anatase phase content estimated according to the Rietveld method fluctuated from 57 ± 0.5 to 64 ± 3 wt %, whereas, for rutile, it was from 6.5 ± 0.2 to 8 ± 1.4 wt %. The diffraction peaks at 30°, 35.6°, 43.3°, 57.3°, 62.9° correspond to [220], [311], [400], [541] and [440] planes of cubic inverse spinel Fe_3_O_4_, respectively [42,43]. The average size of commercial magnetite crystallites estimated using the Scherrer equation for the main peak of magnetite [311] was about 44 ± 1 nm. Moreover, the broad diffraction peak at 2θ = 15–25° corresponded to an amorphous silica layer in the nanocomposite structure [44,45]. For Au-TiO_2_/SiO_2_@Fe_3_O_4_ nanocomposite, the presence of gold nanoparticles (NPs) with diameter of about 21 ± 1 nm was confirmed by diffraction peaks at 64.8° and 77.8° 2θ for Au [220] and [311]. However, other metal nanoparticles were hardly detected on the surface of TiO_2_ indicating either a small amount of deposited metal or a small size of metallic NPs.

**Figure 2:**
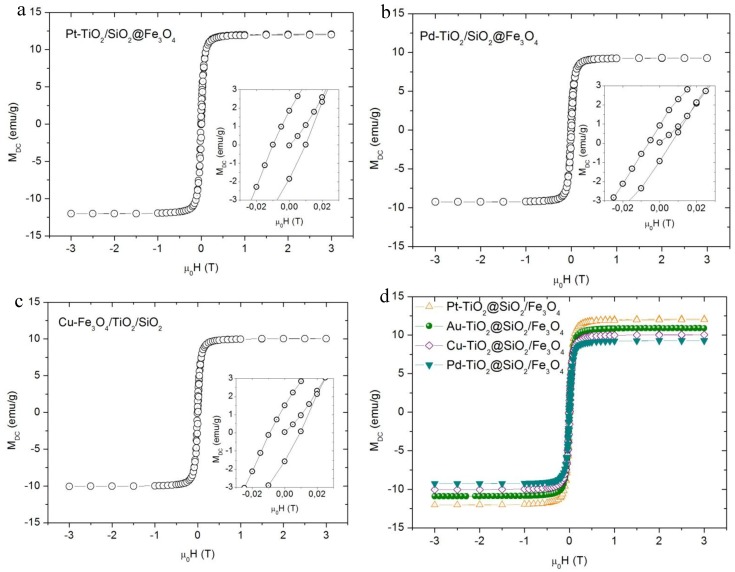
Magnetic hysteresis for (**a**) Pt-TiO_2_/SiO_2_@Fe_3_O_4_; (**b**) Pd-TiO_2_/SiO_2_@Fe_3_O_4_; (**c**) Cu-TiO_2_/SiO_2_@Fe_3_O_4_; (**d**) all obtained Me-TiO_2_/SiO_2_@Fe_3_O_4_ nanocomposites and magnification at hysteresis loop start point.

The magnetic properties of Me-TiO_2_/SiO_2_@Fe_3_O_4_ nanocomposites were measured at room temperature (293 K), and obtained results are presented in Figure 2 and Table 1.

For all obtained photocatalysts, the magnetic saturation reached ca. 10–12 emu·g^−1^ and did not depend on the amount of ferrite fraction in the nanocomposite and noble metal presence at the surface of TiO_2_.

The surface composition of Me-TiO_2_/SiO_2_@Fe_3_O_4_ nanocomposites and oxidation states of C 1s, Ti 2p and Fe 2p were determined by X-ray photoelectron spectroscopy (XPS) analysis, and the obtained data are presented in Table 2.

The main fraction of each nanocomposite was oxygen, as a component of TiO_2_, SiO_2_ and Fe_3_O_4_. The content of oxygen varied from 53.5 to 59.6 at.%. In all Me-TiO_2_/SiO_2_@Fe_3_O_4_ nanocomposites, the peak at binding energy (BE) 289–284 eV attributed to Si 2p was observed. The peak attributed to C 1s was observed at around 284–288 eV. The C 1s region could be deconvoluted for five peaks. It was found that carbon appeared as the COOH (BE~288.8 eV), C=O (BE~287.3 eV), C–OH (BE~285.3 eV) and C–C (aromatic and aliphatic) bonds (BE~283.8 eV and BE~284.3 eV, respectively). Carbon content varied from 9.5 to 13.4 at.% and was observed in the surface layer for all obtained photocatalysts, even for pure TiO_2_: see Table 2. Carbon content originated from the organic precursor of silica (TEOS) and the reaction environment. The correlation between carbon content and UV-visible irradiation-induced activity was not observed. Moreover, the band gaps for TiO_2_ and noble metal modified magnetic photocatalysts were similar and equaled 3.1 eV. Based on energy-dispersive X-ray spectroscopy (EDS) analysis, the carbon content was about 2 wt % in all obtained photocatalysts.

The Ti 2p spectrum could be deconvoluted into two components at 458.6 and 458.1 eV binding energies and could be identified with TiO_2_ and Ti_2_O_3_/Ti^3+^ (in lattice, i.e., “self-doped titania”), respectively [46]. The presence of Ti_2_O_3_ is not expected in well-crystallized titania P25, where even thermal treatment did not cause meaningful phase transition, e.g., an increase in rutile content from 14.4 to 18.8 was observed after 2 h calcination of P25 sample at 500 °C [47]. Intensities of Ti^4+^ and Ti^3+^ components showed that Ti^4+^ was the dominant surface state (96–97%) for all obtained photocatalysts. The presence of all deposited metals was confirmed by XPS analysis, but their presence did not change significantly the surface composition of photocatalyst.

Moreover, the presence of platinum and silver was confirmed by (scanning electron microscopy/energy-dispersive X-ray spectroscopy (SEM/EDS) as is shown in Table 3. It must be pointed that, although metals were detected in all samples by SEM/EDS, their amounts were higher compared to XPS and slightly lower than used for deposition.

**Figure 3:**
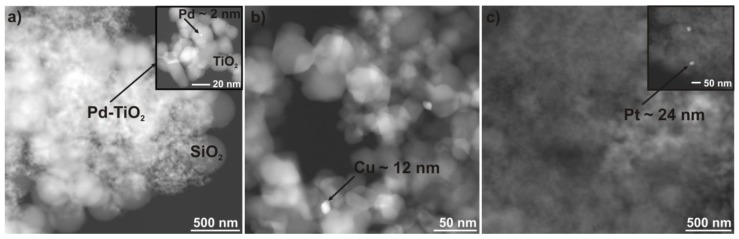
Dark-field scanning transmission (DF-STEM) microscopy images for (**a**) Pd-TiO_2_/SiO_2_@Fe_3_O_4_; (**b**) Cu-TiO_2_/SiO_2_@Fe_3_O_4_ and (**c**) Pt-TiO_2_/SiO_2_@Fe_3_O_4_ nanocomposites. Inserts for (**a**,**c**): magnification of metal nanoparticles deposited on TiO_2_.

Figure 3a–c shows dark-field scanning transmission microscopy (DF-STEM) images of the prepared Pd-TiO_2_/SiO_2_@Fe_3_O_4_, Cu-TiO_2_/SiO_2_@Fe_3_O_4_ and Pt-TiO_2_/SiO_2_@Fe_3_O_4_ photocatalysts. Pd-TiO_2_/SiO_2_@Fe_3_O_4_ nanocomposite had small palladium nanoparticles with a diameter of about 1–2 nm deposited on titania surface, whereas Cu-TiO_2_/SiO_2_@Fe_3_O_4_ contained copper particles with a diameter of about 10–12 nm.

Sample Pt-TiO_2_/SiO_2_@Fe_3_O_4_ had a Pt particle size in the range from 20 to 24 nm deposited on the titania surface. The size of Au nanoparticles of ca. 21 nm determined from XRD patterns was in good agreement with the size obtained from the STEM analysis (24 nm). It is well known that the efficiency of the photocatalytic process depends strongly on the particle size of metal deposits and titania physicochemical properties. It was reported that small and monodisperse silver nanoparticles below 10 nm exhibited the highest photocatalytic and antimicrobial activity [48,49]. However, for gold nanoparticles deposited on titania, an increase in gold NP size resulted in the enhancement of photocatalytic activity, probably due to the ability of the photoabsorption of more photons by larger and rod-like gold nanoparticles of broad plasmonic absorption band [50,51]. On the contrary, a considerable increase of photocatalytic activity was observed for small platinum and palladium NPs (2–3 nm) deposited on TiO_2_ [52]. Therefore, it is assumed that the highest photocatalytic activity should reveal magnetic TiO_2_-based photocatalyst surfaces modified by fine palladium nanoparticles.

**Figure 4:**
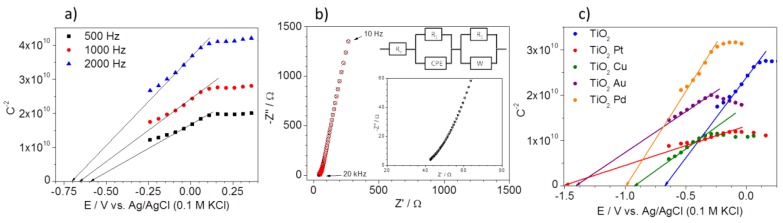
(**a**) Mott-Schottky plot of unmodified photocatalyst (space charge region capacitance calculated for 3 different frequencies); (**b**) Exemplary impedance spectrum and fitted data of unmodified TiO_2_ (recorded at 0 V vs. Ag/AgCl (0.1 M KCl). Insert: equivalent circuit used to model spectrum, and (**c**) comparison of Mott-Schottky plots recorded for TiO_2_, Cu-TiO_2_, Pd-TiO_2_, Au-TiO_2_, and Pt-TiO_2_ (capacitances calculated for 1000 Hz).

Mott-Schottky analysis was used to determine the location of flat band energy (*E*_fb_) by measuring the space charge region capacitance (*C*_sc_) at electrode/electrolyte interface. Exemplary Mott-Schottky plots of Pt/TiO_2_ electrode are presented in Figure 4a. The capacitance of the space charge region was calculated using three different frequencies. As can be seen, different values of *E*_fb_ can be interpolated. The frequency dispersion is observed due to the electrode porosity. An exemplary impedance spectrum with appropriate fitting and used equivalent circuit (EQC) is presented in Figure 4b. The proposed EQC consists of the lowest possible amount of elements.

The capacitance of the semiconductor/electrolyte interphase is fitted using constant phase element (CPE). The diffusional impedance “W” is related to the ionic transport inside the pores of deposited TiO_2_ to the blocking electrode (Pt). The comparison of Mott-Schottky analyses for un-modified and metal-modified TiO_2_ nanocomposites is presented in Figure 4c. The Mott-Schottky plots showed that TiO_2_ acts as an n-type (negative slope) semiconductor in all prepared photocatalysts. The flat band potential of TiO_2_ nanocomposite was estimated at −0.69 V vs. Ag/AgCl (0.1 M KCl) and it is in good agreement with our previous results and literature data [53]. The flat band potentials were −0.925, −1.0, −1.405 and −1.5 V for Cu, Pd, Au and Pt modified nanocomposites, respectively. For all obtained photocatalysts, a significant cathodic shift of the flat band gap potential was observed. Tanabe and Ozaki [54] reported that photocatalytic properties of Me-TiO_2_ photocatalysts depended on the work function of the used metal. Therefore, it can be concluded that the differences of work functions of Cu, Pd, Au, and Pt may also affect the location of flat band potential of modified TiO_2_. Thus, the presence of metal nanoparticles on the surface of titania should enhance the ability of TiO_2_ to oxidize adsorbed species due to efficient electron trapping [55]. The Mott-Schottky plots also demonstrate the difference in the slopes of the curves of TiO_2_ and metal-modified TiO_2_ nanocomposites. There was a significant decrease in the slope of copper-modified TiO_2_ compared to that of TiO_2_, indicating an enhanced charge carrier density and faster charge transfer for Cu-modified TiO_2_, which should contribute to higher photocatalytic activity.

### 2.2. Phenol Photocatalytic Degradation

The photocatalytic activity of the as-prepared nanocomposites was studied by examining the reaction of phenol degradation. No phenol was degraded in the absence of illumination, indicating that there was no dark reaction at the surface of Me-TiO_2_/SiO_2_@Fe_3_O_4_ nanocomposites.

**Figure 5:**
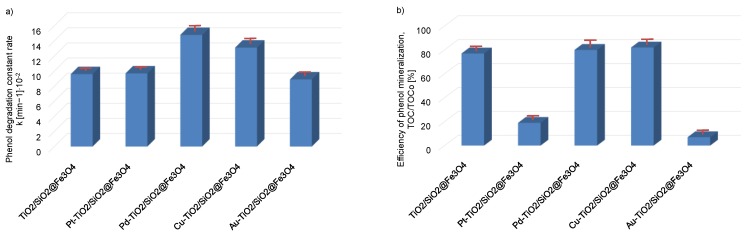
Photodegradation of phenol (**a**) and total organic carbon (TOC) reduction (**b**) under ultraviolet-visible (UV-Vis) light the presence of Pt-TiO_2_/SiO_2_@Fe_3_O_4_, Pd-TiO_2_/SiO_2_@Fe_3_O_4_, Cu-TiO_2_/SiO_2_@Fe_3_O_4_ and Au-TiO_2_/SiO_2_@Fe_3_O_4_ nanocomposites. Experimental conditions: phenol initial concentration Co. = 0.2 × 10^−3^ M, content of photocatalyst 2 g·dm^−3^, 300 W xenon lamp.

Efficiency of phenol photodegration and total organic carbon (TOC) reduction under ultraviolet-visible (UV-vis) light in the presence of titanium(IV) oxide magnetic nanocomposites modified with platinum, palladium, copper or gold nanoparticles are presented in Figure 5a,b, respectively.

The apparent first-order constant rate of phenol photodegradation increased from 8.9 × 10^−2^ ± 0.08 min^−1^ for TiO_2_/SiO_2_@Fe_3_O_4_ and Au-TiO_2_/SiO_2_@Fe_3_O_4_to 14.8 × 10^−2^ ± 0.27 min^−1^ for Pd-TiO_2_/SiO_2_@Fe_3_O_4_. The highest photo-oxidatation rate of phenol was observed for Pd-TiO_2_/SiO_2_@Fe_3_O_4_ and Cu-TiO_2_/SiO_2_@Fe_3_O_4_ photocatalysts. The 20-min irradiation by UV-vis light of Cu-TiO_2_/SiO_2_@Fe_3_O_4_ and Pd-TiO_2_/SiO_2_@Fe_3_O_4_ resulted in 76% and 83% phenol degradation, respectively. The removal rates of TOC for Cu-TiO_2_/SiO_2_@Fe_3_O_4_ and Pd-TiO_2_/SiO_2_@Fe_3_O_4_ were much higher compared to that for Au-TiO_2_/SiO_2_@Fe_3_O_4_ and Pt-TiO_2_/SiO_2_@Fe_3_O_4_ photocatalysts and quite similar to their efficiency of phenol degradation. For the most active Cu-TiO_2_/SiO_2_@Fe_3_O_4_ and Pd-TiO_2_/SiO_2_@Fe_3_O_4_ photocatalysts, the mineralization of phenol, measured as TOC reduction, was about 80 ± 3% and decreased to 19 ± 1% and 7 ± 1% for Pt-TiO_2_/SiO_2_@Fe_3_O_4_ and Au-TiO_2_/SiO_2_@Fe_3_O_4_ nanocomposites, respectively.

The stability of the magnetic particles (i.e., iron oxides) was studied by measuring the photodissolution of the iron oxides using atomic absorption spectroscopy (ASA). As presented in Table 4, the concentration of iron in the reaction medium was below the limit of quantification (1 mg·dm^−3^). The iron content for blank test, i.e., water without photocatalyst, equaled 0.49 ± 0.06 mg·dm^−3^ and in post-reaction medium was about 0.55 ± 0.10. It has been previously reported that combining magnetite and titanium dioxide may lead to the photodissolution of iron oxide phase and the leaching of iron ions to reaction medium [4,5]. However, the introduction of a silica layer results in the prevention of iron oxide photodissolutions [56].

Additionally, a nanocomposite analysis of copper species dissolution for Cu-TiO_2_/SiO_2_@Fe_3_O_4_ was also performed. The content of Cu in post-reaction medium was defined as 0.41 ± 0.08 mg·dm^−3^. The obtained concentration was also below the limit of quantification (0.5 mg·dm^−3^). Therefore, the obtained data clearly indicates that photocatalysts were stable during photocatalytic process and metal leaching was not observed.

### 2.3. Identification of Degradation Intermediates

**Figure 6:**
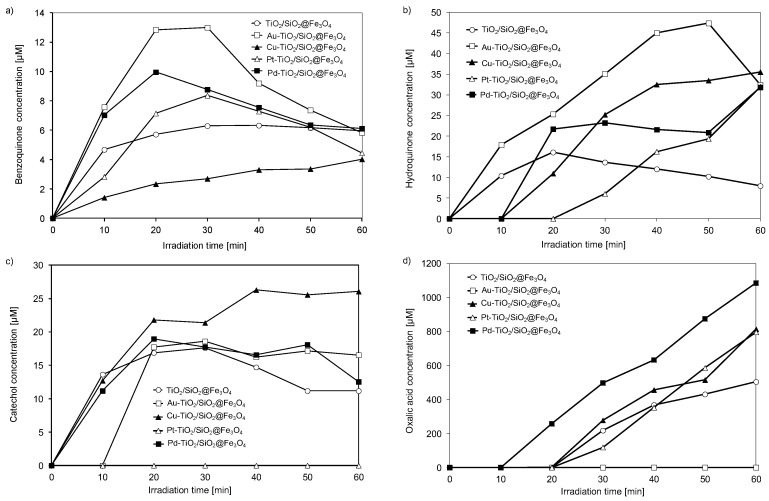
Variation of benzoquinone (**a**); hydroquinone (**b**); catechol (**c**) and oxalic acid (**d**) formation during phenol photodegradation.

Phenol is a non-volatile and common contaminant that is frequently present in industrial wastewaters. The US Environmental Protection Agency (EPA) and the European Union (EU) have classified phenolic compounds as priority pollutants since they are harmful to organisms even at low concentrations. Moreover, a higher content of phenol than 30 mg·dm^−3^ inhibits biological treatment or even eliminates sensitive microorganisms from activated sludge in biological wastewater plants and significantly reduces the biodegradation of other components. Therefore, it is important to study the pathway and intermediates formed during the photo-oxidation of phenol. It is well known that catechol, hydroquinone and 1,4-benzoquinone are the three most important aromatic intermediates in the reaction of phenol degradation [57]. The oxidation route of phenol occurs by the hydroxylation of its molecule to hydroquinone and catechol as a first step with a further oxidation of the dihydroxylbenzenes to benzoquinones [57]. Similarly, in our study, the highest yield was observed for hydroquinone, and then catechol generation during phenol photocatalytic decomposition. As shown in Figure 6, the concentration of hydroquinone and p-benzoquinone in aqueous phase during 60 min of irradiation was much higher for Au-TiO_2_/SiO_2_@Fe_3_O_4_ than that for Pd-TiO_2_/SiO_2_@Fe_3_O_4_ and Cu-TiO_2_/SiO_2_@Fe_3_O_4_ photocatalysts, which revealed a significant enhancement of phenol photomineralization.

Generally, p-benzoquinone can be formed by (1) electrophilic attack of **˙**OH radical, (2) direct photooxidation of phenol by holes h^+^, which may form phenoxyl radical and directly oxidize phenol to benzoquinone, or (3) direct oxidation of hydroquinone by oxygen dissolved in water [57]. Hydroquinone could be produced directly by **˙**OH radical attack on a phenol molecule or by electron e_cb_^−^ reduction of benzoquinone molecule. Interestingly, catechol was not formed during the first 10 min of irradiation only on Au-TiO_2_/SiO_2_@Fe_3_O_4_ nanocomposite. It is proposed that keto-enol tautomeric equilibrium between hydroquinone and benzoquinone acts as a buffer and is responsible for the inhibition of phenol mineralization in the Au-TiO_2_/SiO_2_@Fe_3_O_4_ photocatalytic system [37]. The highest formation of catechol, the lowest amount of formed benzoquinone and lack of hydroquinone generation during first 10 min of irradiation was observed for the Cu-TiO_2_/SiO_2_@Fe_3_O_4_ photocatalyst, whereas, for Pd-TiO_2_/SiO_2_@Fe_3_O_4_ photocatalyst, a high yield of catechol and benzoquinone generation was determined in the reaction mixture. The oxalic acid (aliphatic intermediate) in the reaction mixture appeared after 10 min of irradiation for Pd-TiO_2_/SiO_2_@Fe_3_O_4,_ and after 20 min for Cu-, Pt- and unmodified-TiO_2_/SiO_2_@Fe_3_O_4_, suggesting that the phenyl ring was destroyed, forming carboxylic acid intermediates through photocatalytic degradation. No oxalic acid generation was found for Au-TiO_2_/SiO_2_@Fe_3_O_4_ nanocomposite during 60 min of irradiation.

For Pt-TiO_2_/SiO_2_@Fe_3_O_4_ photocatalyst only benzoquinone was detected in the first 10 min of irradiation, whereas after 20 min of irradiation, hydroquinone and oxalic acid were also formed. However, for platinum-modified photocatalyst, a significant change in the photoactivity with respect to TiO_2_/SiO_2_@Fe_3_O_4_ nanocomposite was not noticed. Therefore, it is proposed that Pt particle sizes were probably decisive for the photocatalytic activity under UV-vis irradiation. Previously, we have reported that platinum deposited on anatase with a particle size below 3 nm exhibited the highest photocatalytic activity. Larger particles (>5 nm diameter) contained a decreased number of surface Pt atoms and therefore revealed decreased activity [48]. Platinum ions interact strongly with TiO_2_ matrix; therefore, the size of titania determines much more the size of platinum than palladium or copper.

**Figure 7:**
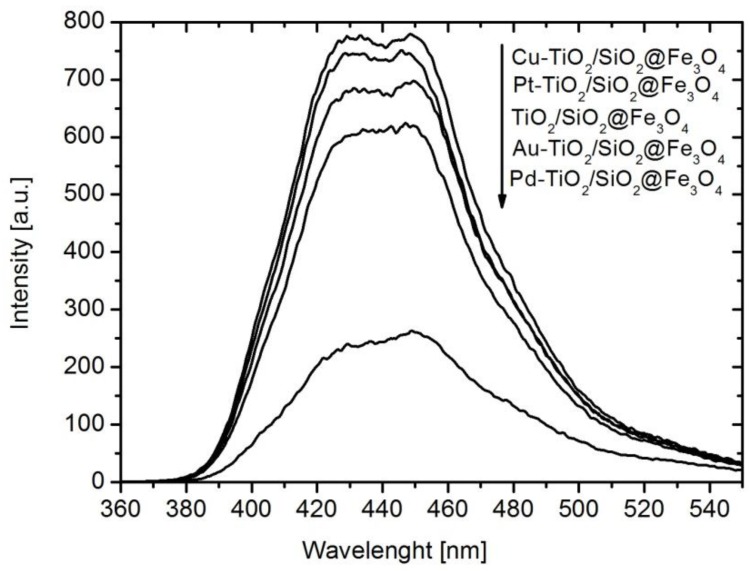
Fluorescence spectra changes observed during irradiation of Me-TiO_2_/SiO_2_@Fe_3_O_4_ photocatalysts under UV-Vis irradiation.

To clarify the mechanism of phenol degradation, the analysis of hydroxyl radical formation on the surface of Me-TiO_2_/SiO_2_@Fe_3_O_4_ photocatalysts under UV-vis irradiation was performed by photoluminescence (PL) spectroscopy using terephtalic acid as a probe molecule. According to Figure 7, it was observed that obtained photocatalysts can produce **˙**OH under UV-vis light irradiation. After 60 min of irradiation, the highest amount of **˙**OH was observed for Cu-TiO_2_/SiO_2_@Fe_3_O_4_ nanocomposite, but modification with Pd resulted in a significant decrease in **˙**OH radical formation (much lower amount than that for unmodified nanocomposite).

The order of **˙**OH radical formation on magnetic photocatalysts was as follows: Cu-TiO_2_/SiO_2_@Fe_3_O_4_ > Pt-TiO_2_/SiO_2_@Fe_3_O_4_ > TiO_2_/SiO_2_@Fe_3_O_4_ > Au-TiO_2_/SiO_2_@Fe_3_O_4_ > Pd-TiO_2_/SiO_2_@Fe_3_O_4_, suggesting that the mechanism of phenol degradation on the Cu-TiO_2_/SiO_2_@Fe_3_O_4_ photocatalyst mainly proceeded by the attack of hydroxyl radicals on the phenyl ring, leading to catechol formation, then being further oxidized to oxalic acid and mineralized to CO_2_. Whereas the obtained results for Pd-TiO_2_/SiO_2_@Fe_3_O_4_ indicated that phenol photocatalytic degradation was attributed to other forms of reactive oxygen species (ROS) e.g., **˙**O_2_^2^^−^, **˙**O_2_^−^, ^1^O_2_ and H_2_O_2_. In order to confirm the role of generated ROS during photocatalytic reaction on the surface of Me-TiO_2_/SiO_2_@Fe_3_O_4_ photocatalysts, the phenol degradation tests were conducted in the presence of e_cb_^−^, h^+^, **˙**O_2_^−^ and **˙**OH scavengers, and under anaerobic conditions (N_2_ purging).

### 2.4. Verification of the Degradation Mechanism Using Scavengers and Under N_2_ Purging

**Figure 8:**
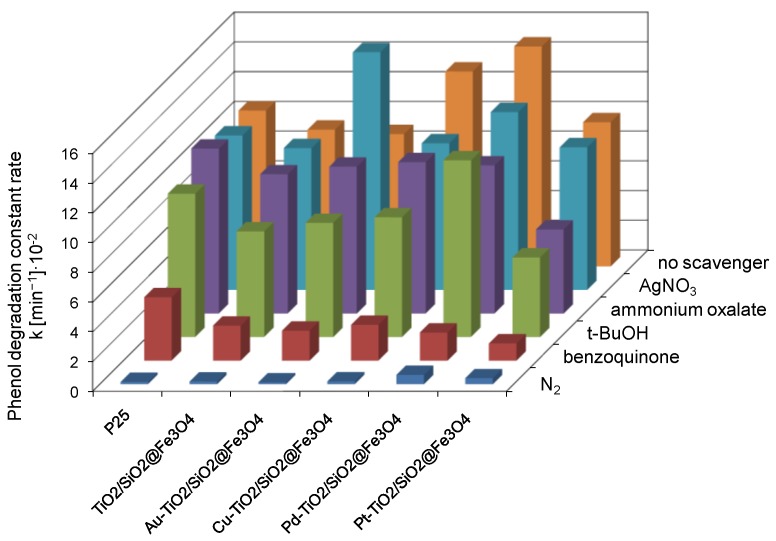
Photocatalytic degradation of phenol for Me-TiO_2_@SiO_2_/Fe_3_O_4_ photocatalysts in the presence of e^−^, h^+^, **˙**O_2_^−^ and **˙**OH scavengers and N_2_ purging.

The active species were investigated to understand the photocatalytic reaction mechanism. The holes (h^+^), hydroxyl radicals (**˙**OH) and superoxide radical anion (**˙**O_2_^−^) are the probable active species taking part in the photodegradation of organic pollutants. Results of the photocatalytic activity in reaction of phenol degradation in the presence of e^−^, h^+^, **˙**O_2_^−^ and **˙**OH scavengers, i.e., silver nitrate, ammonium oxalate, benzoquinone and tert-butyl alcohol, respectively, and during N_2_ purging are presented in Figure 8.

The degradation constant rates, determined without scavengers, serve as reference materials for particular photocatalysts. In the presence of no scavenger, the highest activity was exhibited by Pd-TiO_2_/SiO_2_@Fe_3_O_4_ and Cu-TiO_2_/SiO_2_@Fe_3_O_4_ photocatalysts. The lowest activity was found for SiO_2_@Fe_3_O_4_, with a degradation constant rate of 0.84 × 10^−2^ min^−1^. Compared to metal-modified TiO_2_/SiO_2_@Fe_3_O_4_ nanoparticles, the activity of silica-magnetite nanocomposite was negligible, and therefore no further experiments were carried out for this sample. The low activity of SiO_2_@Fe_3_O_4_ indicates that, despite the presence of ferric ions in the structure of nanocomposite, the photocatalytic activity results only from the presence of TiO_2_ on the surface of magnetic nanocomposite, whereas SiO_2_@Fe_3_O_4_ plays the role for an inert structure.

#### 2.4.1. Effect of N_2_ Purging

Molecular oxygen dissolved in water solution acts as an electron acceptor in photodegradation process, which limits the recombination rate between electron and hole through the formation of reactive oxygen species. In this study, the effect of dissolved oxygen was investigated by N_2_ purging. As shown in Figure 8, the decrease concentration of oxygen in reaction solution resulted in inhibition of phenol degradation for all obtained magnetic photocatalysts. This observation demonstrates the predominant role of reactive oxygen species in mechanism of phenol degradation.

#### 2.4.2. Effect of Benzoquinone

To identify the contribution of superoxide radical species on the photocatalytic degradation, prior to irradiation 500 mg·dm^−3^ of benzoquinone (BQ) was added into phenol solution. The photocatalytic performance was significantly suppressed after BQ was introduced, indicating that **˙**O_2_^−^ played a crucial role in the photodegradation process. Phenol degradation efficiency was reduced by 88% for Pt-TiO_2_/SiO_2_@Fe_3_O_4_ and Pd-TiO_2_/SiO_2_@Fe_3_O_4_ photocatalysts. For Au-TiO_2_/SiO_2_@Fe_3_O_4_, Cu-TiO_2_/SiO_2_@Fe_3_O_4_ and TiO_2_/SiO_2_@Fe_3_O_4_ nanocomposites, the addition of BQ inhibited the photocatalytic efficiency by 75–77% and slightly lower for TiO_2_ P25 (60%).

#### 2.4.3. Effect of t-BuOH

The tert-butyl alcohol (t-BuOH) was utilized to trap the photogenerated hydroxyl radical (**˙**OH). The introduction of t-BuOH resulted in decrease of phenol degradation constant rate mainly for Pt-TiO_2_/SiO_2_@Fe_3_O_4_ and Cu-TiO_2_/SiO_2_@Fe_3_O_4_ nanocomposites. The degradation constant rate was 45% and 38% lower than without tert-butyl alcohol and equalled 5.36 × 10^−2^ and 8.06 × 10^−2^ min^−1^ for Pt-TiO_2_/SiO_2_@Fe_3_O_4_ and Cu-TiO_2_/SiO_2_@Fe_3_O_4_, respectively. Less affected by t-BuOH scavenger was Pd-TiO_2_/SiO_2_@Fe_3_O_4_ magnetic photocatalyst and TiO_2_ P25. A decrease in phenol degradation constant rate from 14.8 × 10^−2^ to 11.97 × 10^−2^ min^−1^ was observed for Pd-TiO_2_/SiO_2_@Fe_3_O_4_ and from 10.49 × 10^−2^ to 9.65 × 10^−2^ min^−1^ for TiO_2_ P25.

#### 2.4.4. Effect of Ammonium Oxalate

Ammonium oxalate was introduced as the scavenger of photogenerated holes (h^+^). The photodegradation reaction was partly suppressed by 33% and 42% for Pd-TiO_2_/SiO_2_@Fe_3_O_4_ and Pt-TiO_2_/SiO_2_@Fe_3_O_4_ nanocomposites, suggesting that h^+^ also played a role in the process of phenol oxidation. For Cu-TiO_2_/SiO_2_@Fe_3_O_4_, Au-TiO_2_/SiO_2_@Fe_3_O_4_, TiO_2_/SiO_2_@Fe_3_O_4_ and TiO_2_ P25 photocatalysts, ammonium oxalate capturing of h^+^ have little or almost no effect on the efficiency of phenol degradation. Thus, h^+^ is not the important active species for the reaction of phenol degradation in the presence of P25, TiO_2_/SiO_2_@Fe_3_O_4_ and TiO_2_/SiO_2_@Fe_3_O_4_ modified by copper or gold nanoparticles.

#### 2.4.5. Effect of Silver Nitrate

Silver nitrate was utilized to trap the photogenerated electrons. The phenol degradation for TiO_2_ P25, Cu-TiO_2_/SiO_2_@Fe_3_O_4_, Pt-TiO_2_/SiO_2_@Fe_3_O_4_ and Pd-TiO_2_/SiO_2_@Fe_3_O_4_ photocatalysts in the presence of AgNO_3_ caused a negligible decrease in the photocatalytic efficiency. Moreover, for TiO_2_/SiO_2_@Fe_3_O_4_ and Au-TiO_2_/SiO_2_@Fe_3_O_4_ photocatalysts, the addition of silver ions has a rate-enhancing effect. Ag^+^ ion could trap photogenerated electrons to avoid the recombination of electrons and holes. The positive effect of electron trapping may also result from the in-situ formation of bimetallic particles of Ag-Au deposited on the surface of TiO_2_/SiO_2_@Fe_3_O_4_ nanocomposite. It was reported that such coupling of Ag and Au increases the photocatalytic performance of the photocatalyst [58,59].

These results confirm the crucial role of reactive oxygen species (**˙**O_2_^−^ and **˙**OH) in the photocatalytic degradation of phenol in the presence of Me-TiO_2_/SiO_2_@Fe_3_O_4_ photocatalysts.

## 3. Discussion and Proposed Mechanism

The degradation of organic pollutants proceeds through the formation of intermediates, which often are very stable and toxic for the environment. In order to do the above, it is essential to investigate the mechanism of pollutant degradation. Of particular interest should be studies on correlation between activity, composition and selectivity of photocatalysts.

Oxidation or reduction pathways of TiO_2_-based photocatalytic reaction may depend on the crystalline structure, surface or bulk modification of semiconductor, pH and salinity of the surrounded medium [60,61,62].

The first step of photocatalytic reaction involves excitation of TiO_2_. Photogenerated hole and electron pairs may recombine or migrate on the semiconductor surface, taking part in reaction with water, oxygen and other species present on the TiO_2_ surface or in the surrounded medium. However, the adsorbed molecules may be oxidized via direct or indirect methods [63]. A direct oxidation proceeded through the reaction of free holes/electrons with adsorbed organics, while an indirect oxidation proceeds through oxidation by reactive oxygen species (ROS). ROS are formed, depending on the pH of surrounding solution, by reaction of photogenerated holes with terminal oxygen ions or terminal hydroxyl groups. ROS may be also generated during a recombination of organic radicals. In our study, the pH of the aqueous phase was slightly basic, indicating (pH > pH_IEP_) formation mainly of superoxide radicals. Miyazaki et al. [64] observed that, at room temperature, the generation of dihydroxybenzenes is favored during both direct and indirect oxidation. Kim and Choi [35] observed that photocatalytic activity of phenols may be also affected by the formation of surface complexes of adsorbed molecule and terminal titanium atoms. They proposed that the complex formation included covalent bonding as well as physical adsorption and depended on the surface area affected by TiO_2_ structure. The complex formation could induce visible light activity, whereas, under UV light, hydroxyl radical generation played the main role. The formation of TiO_2_-organic molecule complex was observed for bare titanium(IV) oxide P25, while for Pt-TiO_2_ and F-TiO_2_ the complexation did not occur, due to blocking Ti-OH sites and the changing acidity of OH groups. Murcia et al. [65] have also investigated the formation of surface complex on Pt-TiO_2_ surface. They observed that platinum nanoparticles favored the adsorption of phenol on photocatalyst surface by the formation of bidentate phenolates species. The adsorption depended on platinum NP size and the oxidation state of Pt species [65]. Small palladium nanoparticles preferentially are formed on lattice defects. Similarly, our previous studies for Ag/Au-, Pt/Pd- and Ag/Pt-modified TiO_2_ showed the dependence of particle size of platinum and gold NPs on the crystallite size of titania particles, where small anatase with a larger number of surface defects than that in well crystallized larger particles of rutile and anatase stimulated formation of fine noble metal nanoparticles [48,59,66].

**Figure 9:**
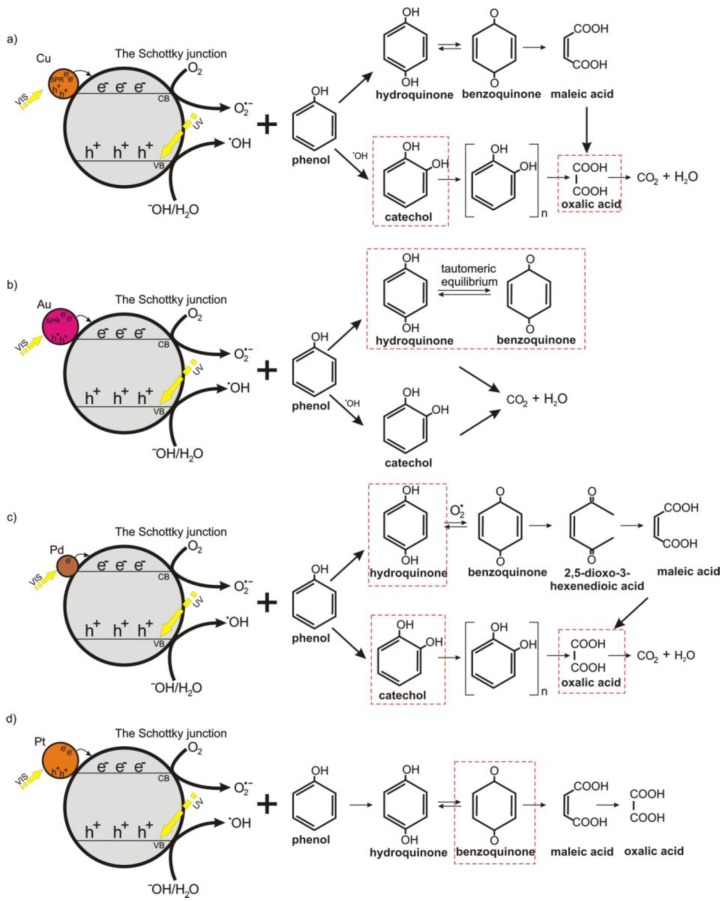
Schematic illustration of the phenol photocatalytic degradation mechanism for magnetic photocatalysts modified with: (**a**) copper; (**b**) gold; (**c**) palladium and (**d**) platinum nanoparticles.

Combining the obtained results, a possible mechanism for the photocatalytic degradation of phenol with metal-modified (Me = Pd, Pt, Cu, Au) TiO_2_/SiO_2_@Fe_3_O_4_ photocatalysts is proposed and illustrated in Figure 9. For Cu-TiO_2_/SiO_2_@Fe_3_O_4_, superoxide and hydroxyl radicals are mainly active species involved in phenol degradation, which attack the phenyl ring yielding catechol, and hydroquinone generation (benzoquinone was also detected but at significantly lower concentration). Then, the phenyl rings in these compounds disintegrate and short-chain organic acids are produced, mainly oxalic acid, which further mineralized to CO_2_ and H_2_O (see Figure 9a). For Au-TiO_2_/SiO_2_@Fe_3_O_4_, the surface plasmon resonance induces electrons to be transferred to the conduction band of TiO_2_ and are trapped by dissolved oxygen, resulting in generation of **˙**O_2_^−^. The main intermediates determined in the first minutes of irradiation are benzoquinone and hydroquinone. However, the induction period was observed for generation of catechol by oxidation of phenol in the presence of h_vb_^+^. As shown in Figure 9b, the keto-enol tautomeric equilibrium between hydroquinone and benzoquinone retards the rate of phenol photomineralization.

The mechanism of phenol degradation in the presence of Pd-TiO_2_/SiO_2_@Fe_3_O_4_ photocatalyst proceeds by generation of reactive oxygen species, e.g., **˙**O_2_^−^, which oxidize phenol to benzoquinone, hydroquinone and catechol (see in Figure 9c). The highest concentration of benzoquinone and hydroquinone was observed at the beginning of the reaction. However, hydroquinone concentration decreased slower compared to the catechol amount due to different pathways of phenol photo-oxidation. Our observations are in good agreement with literature. Santos et al. [67] found that catechol oxidation did not yield either benzoquinone or maleic acid formation, but oxalic acid, which finally was mineralized to CO_2_. However, benzoquinone and maleic acid are products, which are determined in the hydroquinone oxidation. The induction period for hydroquinone oxidation was reported, meaning that fewer or more-quickly oxidizable intermediates are produced from catechol than from hydroquinone [67]. In our study, a much higher mineralization rate was observed for Pd-TiO_2_/SiO_2_@Fe_3_O_4_ and Cu-TiO_2_/SiO_2_@Fe_3_O_4_ photocatalysts, which produced the highest concentration of catechol during photocatalytic reaction. For Pt-TiO_2_/SiO_2_@Fe_3_O_4_ nanocomposite, catechol was not detected after 60 min of irradiation, resulting in a low oxidation to oxalic acid and mineralization to CO_2_ (see in Figure 9d). Therefore, it is proposed that enhanced activity is related to a decrease in palladium particle size, an increase in adsorption sites and efficient separation of charge carriers.

## 4. Materials and Methods

### 4.1. Materials and Instruments

Commercial TiO_2_ P25 (a mixture of the crystalline phases: anatase (73–85%), rutile (14–17%), and amorphous titania (0–13%) [1,2], Brunauer-Emmett-Tellersurface area S_BET_ = 50 m^2^·g^−1^ supplier: Evonik, Essen, Germany) was used for the preparation of Me-TiO_2_/SiO_2_@Fe_3_O_4_ photocatalysts. Ferrous ferric oxide (Fe_3_O_4_, 97%) with particle size of about 50 nm was purchased from Aldrich (St. Louis, MO, USA) and used as a magnetic core of obtained Me-TiO_2_/SiO_2_@Fe_3_O_4_ nanocomposites. Ethyl alcohol (99.8%) was used as solvent and tetraethyl orthosilicate (TEOS, Si(OC_2_H_5_)_4_), 99%), provided by Aldrich (St. Louis, MO, USA), was used as starting material for the preparation of SiO_2_@Fe_3_O_4_ nanoparticles. Ammonium hydroxide solution (25%) was purchased from Avantor (Center Valley, PA, USA). Gold(III) chloride, copper(II) nitrate, palladium(II) chloride, chloroplatinic acid hexahydrate and sodium borohydride were provided by Aldrich and used as starting materials for preparation of metallic nanoparticles (NPs) deposited on TiO_2_/SiO_2_@Fe_3_O_4_. Phenol (99.5%) was provided by Aldrich (St. Louis, MO, USA) and used as model organic pollutant. Intermediates of photocatalytic phenol decomposition e.g., benzoquinone, hydroquinone, catechol, maleic acid and oxalic acid were purchased from Fluka (Shanghai, China) and used for quantitative determination of their concentration during photodegradation process. Charge carrier scavengers, such as ammonium oxalate, silver nitrate, butyl alcohol and benzoquinone were provided by Aldrich (St. Louis, MO, USA). All reagents were used without further purification.

XRD analysis was performed using a Rigaku Intelligent X-ray diffraction system (SmartLab, Neu-Isenburg, Germany) equipped with a sealed tube X-ray generator (Neu-Isenburg, Germany). Measurements were performed on the 2θ range of 10–80° with the scan speed of 1.00°·min^−1^ and scan step of 0.01°. Crystallite size of photocatalysts in the direction vertical to the corresponding lattice plane was determined using the Scherrer equation, based on the corrected full width at half maximum (FWHM) of the XRD peak and angle of diffraction.

To characterise the light-absorption properties of modified photocatalysts, diffuse reflectance (DR) spectra were recorded, and data were converted to obtain absorption spectra. To characterise the light-absorption properties of modified photocatalysts, diffuse reflectance (DR) spectra were recorded, and data were converted to obtain absorption spectra. The band gap energy of photocatalysts was calculated from the corresponding Kubelka-Munk function, $F{\left(R\right)}^{0.5}E_{\mathrm{ph}}^{0.5}$ against $E_{\mathrm{ph}}$, where $E_{\mathrm{ph}}$ is photon energy. The measurements were carried out on ThemoScientific (Waltham, MA, USA) evolution 220 spectrophotometer equipped with PIN-757 integrating sphere (Waltham, MA, USA). Commercial TiO_2_ P25 was used as a reference sample.

XPS analysis was carried out in multichamber ultrahigh vacuum (UHV) system (Omicron nanoTechnology, Taunusstein, Germany), at room temperature in a ultra-high vacuum conditions, below 1.1 × 10^−8^ mBar. The photoelectrons were excited by an Mg-K_α_ X-Ray source. The X-ray anode was operated at 15 keV and 300 W. Omicron Argus hemispherical electron analyser with round aperture of 4 mm was used for analysis of emitted photoelectrons. Measurements were carried out in a constant analyser energy (CAE) mode with pass energy equal 50 eV. The binding energies were corrected using the background C 1s line (285.0 eV) as a reference. XPS spectra were analysed with Casa-XPS software (Prevac, Rogów, Poland) using a Shirley background subtraction and Gaussian-Lorentzian curve as a fitting algorithm.

The particle size, dispersion uniformity and chemical composition of obtained nanocomposites was examined using transmission electron microscopy (HRTEM) using FEI Europe, Tecnai F20 X-Twin (Waltham, MA, USA).

Magnetic hysteresis loops were carried out using psychical properties measurements system (PPMS, Quantum Design, San Diego, CA, USA). Measurements were performed at temperature of 293 K, in the range of 0–30,000 Oe.

Electrochemical impedance spectroscopy (EIS) measurements were performed using the potentiostat-galvanostat AutoLab PGStat302N system (Utrecht, The Netherlands) under GPES/FRA software control. Electrochemical experiments were performed in a three-electrode cell in 0.2 M K_2_SO_4_ in pH adjusted to 7. Ag/AgCl (0.1 M KCl) was used as a reference electrode and platinum mesh acted as a counter electrode. The impedance spectra were taken at the frequency range from 10 kHz to 100 Hz with 10 mV amplitude of the alternating current.

AC signal. Space charge region capacitances have been calculated from single frequency (1000 Hz) using Equation (1).

(1) $$C_{\mathrm{sc}}=-\frac{1}{Z\prime \prime \omega }$$

*C*—capacitance, *Z*ʺ—imaginary impedance, *ω*—angular frequency.

Mott-Schottky analysis was performed in order to examine the influence of metal nanoparticles on the flat band potential of metal-modified TiO_2_. Flat band potential can be estimated from intercept of *C*_sc_^−2^ vs. *E*, according to Equation (2) assuming that the term k_B_*T*/*e* is small and can be neglected, where

(2) $$C_{\mathrm{sc}}^{-2}=\left(\frac{2}{{\varepsilon \varepsilon }_{0}{\mathrm{eN}}_{D}}\right)\left(E-E_{fb}-\frac{k_{B}T}{e}\right)$$

*C*_sc_—capacitance of space charge region, *ε* and *ε*_0_—dielectric constant of the material and permittivity of free space, e—electronic charge, N_D_—the number of donors, *E*—applied potential, *E*_fb_—flat band potential, k_B_—Boltzmann’s constant, *T*—temperature.

Impedance spectra analysis and fitting were performed using EIS Spectrum Analyzer software.

Atomic absorbance spectroscopy (AAS) measurements were performed using SENS AA DUAL spectrometer (GBC EQUIPMENT, Hampshire, IL, USA) equipped with hollow cathodic lamps Cu (GBC EQUIPMENT) and Fe (CPI International, Palo Alto, CA, USA).

### 4.2. Preparation of TiO_2_/SiO_2_@Fe_3_O_4_ and Me-TiO_2_/SiO_2_@Fe_3_O_4_ Photocatalysts

TiO_2_/SiO_2_@Fe_3_O_4_ nanocomposites were prepared by an ultrasonic-assisted sol-gel method. Firstly, 1 g of commercial Fe_3_O_4_ magnetic nanoparticles was dispersed in 50 cm^3^ of ethanol and ultrasonicated for 15 min. Subsequently, 100 cm^3^ of ethanol and 20 cm^3^ of water were added to the suspension and sonicated for 30 min. Then, 150 cm^3^ of ammonia ethanolic solution was dropwise added into magnetite dispersion and sonicated for another 15 min. In the next step, 7.71 cm^3^ of tetraethyl orthosilicate (TEOS), preliminarily diluted in ethanol, was added to the magnetite particles suspension and ultrasonicated for the next 15 min. The weight ratio of magnetite to titanium(IV) oxide was equal to 1:2, whereas molar ratios of TEOS to Fe_3_O_4_ and NH_4_OH to TEOS were 8:1 and 16:1, respectively. After aging of silica gel the suspension of commercial TiO_2_ (P25) in 50 cm^3^ of ethanol was added into SiO_2_@Fe_3_O_4_ dispersion and stirred for 2 h. The obtained suspension of TiO_2_/SiO_2_@Fe_3_O_4_ photocatalyst was separated, dried at 70 °C to dry mass and calcined at 400 °C for 2 h.

In order to obtain metal modified TiO_2_ on SiO_2_@Fe_3_O_4_ nanoparticles, 1.25 cm^3^ of 0.1 M aqueous solution of particular metal precursor (Cu, Au, Pt, and Pd) was added dropwise into the suspension of TiO_2_/SiO_2_@Fe_3_O_4_ photocatalyst and stirred for 30 min. The amount of deposited metal on TiO_2_ was established to be 0.5 mol % regarding the amount of TiO_2_. Metal ions were reduced by adding of 0.1 M aqueous solution of sodium borohydride. The molar ratio of NaBH_4_ to metal ions equaled to 1.5. The obtained suspension of Me-TiO_2_/SiO_2_@Fe_3_O_4_ photocatalyst was centrifuged, dried at 70 °C to dry mass and calcined at 400 °C for 2 h.

Preparation of samples of Au-–TiO_2_@SiO_2_/Fe_3_O_4_ and Cu-TiO_2_@SiO_2_/Fe_3_O_4_ were repeated three times, and preparation of Pt-TiO_2_@SiO_2_/Fe_3_O_4_ was repeated two times without any changes in physicochemical properties (XRD, BET, colour) and photocatalytic activity.

### 4.3. Measurements of Photocatalytic Activity

Photocatalytic activities of the obtained samples were estimated by measurement of the rate of phenol decomposition in an aqueous solution under UV-vis irradiation. For each experiment, 50 mg of the photocatalyst was suspended in 25 cm^3^ of aqueous solution of phenol (Co. = 20 mg·dm^−3^). The obtained suspension was mixed in darkness for 30 min to provide uniform adsorption of phenol on photocatalyst surface, and then irradiated under continuously stirring using 300-W xenon lamp of 50-mW·cm^−2^ power flux. Aliquots of 1.0 cm^3^ of the aqueous suspension were collected after equal time invertals of irradiation, and filtered through syringe filters (*ϕ* = 0.2 m) to remove the photocatalyst particles. The temperature of the aqueous phase during irradiation was kept at 20 °C using a water bath. Phenol concentration was estimated by colorimetric method using a UV-vis spectrophotometer (Thermo Evolution 220). Phenol and phenol degradation intermediates were determined chromatographically using HPLC system (Kyoto, Japan) with UV-vis detector Shimadzu SPD-6A (detection wavelength: 254 nm) (Kyoto, Japan) and a WAKOSIL-II 5C18 AR column (dimensions 4.6 × 250 mm) (Wako Pure Chemical Industries, Tokyo, Japan) with a mobile phase contained water, acetonitrile and phosphoric acid with volume ratio of 70:29.5:0.5, respectively. Photocatalytic degradation analysis were repeated at least three times for each obtained magnetic photocatalyst.

The effect of charge carrier scavengers was examined by addition into the phenol solution (before introduction of the photocatalyst) 1 cm^3^ of 500 mg·dm^−3^ of tert-butyl alcohol (t-BuOH), ammonium oxalate ((NH_4_)_2_C_2_O_4_), silver nitrate (AgNO_3_) or benzoquinone (BQ).

The formation of hydroxyl radicals in suspension of Me-TiO_2_/SiO_2_@Fe_3_O_4_ photocatalyst under UV-vis irradiation was evaluated by photoluminescence (PL) spectroscopy using terephthalic acid as a probe molecule under alkaline conditions. Hydroxyl radicals, produced during photocatalytic process, reactedwith terephtalic acid (TA) generating 2-hydroxyterephtalic acid, which emited fluorescence at around 426 nm [68]. Formation of hydroxyl radicals was estimated using the same experimental set-up as for measuring the decomposition rate of phenol under UV-vis light. After irradiation, the solution was filtered and analyzed on a Perkin Elmer LS55 (Waltham, MA, USA) fluorescence spectrophotometer with an excitation wavelength of 315 nm using NG3 and NG5 (Opole, Poland) cut-off filters. The spectra were recorded in the range of 360–550 nm.

## 5. Conclusions

The preparation procedure and characterization of new metal-modified (Me = Pd, Au, Pt, Cu) TiO_2_/SiO_2_@Fe_3_O_4_ nanocomposites was reported. XPS analysis revealed that the deposition of different metals changed the surface composition of photocatalysts. The highest content of oxygen vacancies (Ti^3+^) was observed for Pd-TiO_2_/SiO_2_@Fe_3_O_4_ and Cu-TiO_2_/SiO_2_@Fe_3_O_4_ nanocomposites. For all obtained photocatalysts, the magnetic saturation was about 10–12 emu·g^−1^ and did not depend on the amount and kind of metal deposited on the surface of TiO_2_. Mott-Schottky analysis showed a significant decrease in the slope of copper-modified TiO_2_ compared to that of TiO_2_, indicating an enhanced charge carrier density and faster charge transfer for Cu-modified TiO_2_ nanoparticles. The highest photooxidatation rate of phenol and mineralization, measured as TOC reduction, was observed for Pd-TiO_2_/SiO_2_@Fe_3_O_4_ and Cu-TiO_2_/SiO_2_@Fe_3_O_4_ photocatalysts. Based on fluorescence spectra and analysis of scavenger formation, it has been found that superoxide and hydroxyl radicals are main active species involved in the degradation, which attacks the phenyl ring yielding catechol, hydroquinone and benzoquinone generation, followed by oxalic acid and CO_2_ formation. It was found that the pathways for hydroquinone and catechol oxidation were different. Catechol was directly oxidized to oxalic acid and then mineralized to CO_2_, while the pathway of degradation for hydroquinone proceeded through the formation of a larger amount of intermediates, e.g., benzoquinone, maleic acid, which were further oxidized to aliphatic carboxylic acids and finally to CO_2_. The highest concentration of catechol and then oxalic acid during photocatalytic reaction was observed for the most active Pd-TiO_2_/SiO_2_@Fe_3_O_4_ and Cu-TiO_2_/SiO_2_@Fe_3_O_4_ photocatalysts. The enhanced activity was related to a decrease in noble metal and semi-noble metal particle size, an increase in the adsorption sites and efficient separation of charge carriers. For Au-TiO_2_/SiO_2_@Fe_3_O_4_ the keto-enol tautomeric equilibrium retarded the rate of phenol photomineralization. Therefore, it is proposed that the hydroquinone pathway is a limiting step for phenol degradation.

## Figures and Tables

**Table 1:** Characteristic of physicochemical properties of TiO_2_/SiO_2_@Fe_3_O_4_ and Me-TiO_2_/SiO_2_@Fe_3_O_4_ photocatalysts.

Photocatalyst	Crystalline Size						Magnetization (emu·g^−1^)
	TiO_2_				Magnetite		
	Anatase		Rutile				
	Size (nm)	Phase Content wt %	Size (nm)	Phase Content wt %	Size (nm)	Phase Content wt %	
TiO_2_/SiO_2_@Fe_3_O_4_	20	57 ± 0.5	30	8 ± 1	44.5	34 ± 1	11
Pt-TiO_2_/SiO_2_@Fe_3_O_4_	19.5	63 ± 2.5	28	6.5 ± 0.2	44	29 ± 2	12
Pd-TiO_2_/SiO_2_@Fe_3_O_4_	19	64 ± 3	31	8 ± 1.4	43	29 ± 2.5	9.5
Cu-TiO_2_/SiO_2_@Fe_3_O_4_	20	61 ± 3	30	7.5 ± 0.5	44	30 ± 3	10
Au-TiO_2_/SiO_2_@Fe_3_O_4_	20	60 ± 1	31	7 ± 0.8	42	32 ± 2	11

**Table 2:** X-ray photoelectron spectroscopy (XPS) analysis of oxygen, titanium, carbon and metal for Me-TiO_2_/SiO_2_@Fe_3_O_4_ photocatalysts, and fraction of oxidation states from deconvolution of XPS peaks of Ti 2p and C 1s.

Photocatalyst	Content (at.%)					Ti 2p_3/2_ (%)		C 1s (%)				
	Ti 2p	O 1s	Si 2p	C 1s	Metal	Ti^4+^	Ti^3+^	C-C	C-O	C=C	C=O	COOH
TiO_2_/SiO_2_@Fe_3_O_4_	8.7	60.0	20.4	10.9	0	97.0	3.0	34.0	24.5	12.8	18.1	10.5
Pd-TiO_2_/SiO_2_@Fe_3_O_4_	8.3	59.4	20.5	11.6	0.2	96.0	4.0	33.9	21.5	22.0	11.5	9.0
Cu-TiO_2_/SiO_2_@Fe_3_O_4_	6.7	56.5	22.6	13.4	0.8	96.0	4.0	43.1	41.2	8.4	2.9	4.4
Au-TiO_2_/SiO_2_@Fe_3_O_4_	9.8	57.6	19.1	13.3	0.2	97.0	3.0	31.5	43.4	18.8	3.0	3.3
Pt-TiO_2_/SiO_2_@Fe_3_O_4_	8.5	59.6	22.3	9.5	0.1	97.0	3.0	41.1	35.5	7.4	15.4	0.6

**Table 3:** XPS and scanning electron microscopy/ energy-dispersive X-ray spectroscopy (SEM/EDS) results of the noble metal content in the magnetic photocatalysts.

Photocatalyst	Amount of Metal		
	Used for Deposition (mol %)	Deposited	
		EDS (wt %)	XPS (at.%)
TiO_2_/SiO_2_@Fe_3_O_4_	0	0	0
Pd-TiO_2_/SiO_2_@Fe_3_O_4_	0.5	0.5	0.2
Cu-TiO_2_/SiO_2_@Fe_3_O_4_	0.5	0.7	0.8
Au-TiO_2_/SiO_2_@Fe_3_O_4_	0.5	0.49	0.2
Pt-TiO_2_/SiO_2_@Fe_3_O_4_	0.5	0.58	0.1

**Table 4:** Iron leaching to reaction medium.

Photocatalysts	Iron Concentration (mg·L^−^^l^)	Comment
TiO_2_/SiO_2_@Fe_3_O_4_	0.55 ± 0.08	<LOQ
Cu-TiO_2_/SiO_2_@Fe_3_O_4_	0.53 ± 0.07	<LOQ
Pd-TiO_2_/SiO_2_@Fe_3_O_4_	0.50 ± 0.08	<LOQ
Pt–TiO_2_/SiO_2_@Fe_3_O_4_	0.53 ± 0.08	<LOQ
Au–TiO_2_/SiO_2_@Fe_3_O_4_	0.55 ± 0.10	<LOQ
